# Pre-exposure to Tempting Food Reduces Subsequent Snack Consumption in Healthy-Weight but Not in Obese-Weight Individuals

**DOI:** 10.3389/fpsyg.2018.00685

**Published:** 2018-05-25

**Authors:** Angelos Stamos, Hannelore Goddyn, Andreas Andronikidis, Siegfried Dewitte

**Affiliations:** ^1^Faculty of Business Economics, KU Leuven, Leuven, Belgium; ^2^Department of Business Administration, University of Macedonia, Thessaloniki, Greece

**Keywords:** healthy-weight, obese-weight, pre-exposure, consumption, tempting food

## Abstract

Obesity has become a severe worldwide problem. Compared to healthy-weight individuals, obese individuals seem to show an increased sensitivity to tempting food. In the present study, we test the pre-exposure effect, which implies that consumption of tempting food is decreased after exposure to tempting food cues in a context of a task that discourages food consumption. Healthy-weight and obese-weight participants were recruited via social media and university channels. Participants took part in a scrabble task with either candy letters or foam letters and subsequently engaged in a taste test. Results showed that in healthy-weight participants, consumption was reduced after solving the scrabble task with candy letters in comparison to foam letters. In obese-weight participants, consumption was reduced in the condition using foam letters (in comparison with healthy-weight participants). The pre-exposure effect was replicated in healthy-weight participants, but could not be observed in participants with obesity, since consumption was reduced in general in this group. Our results suggest that more work should be done to understand how food nudges work in the context of obesity.

## Introduction

Obesity has become a severe worldwide problem and prevalence rates have almost doubled since the eighties and are still increasing (World Health Organization, 2016). The societal relevance of the phenomenon has inspired much research, including behavioral research investigating strategies to improve people’s eating behaviors. The behavioral change strategies resulting from this research are mainly focusing on education, reducing the access to food temptations, or the training of inhibitory control. These tools are very valuable, but do not seem to arm people sufficiently against food in our so-called “obesogenic” environment (Appelhans et al., 2016). Appelhans et al. (2016) point out that exactly during the episodes where temptations pull hard, education and inhibitory control tend to lose their grip. One important reason for the moderate effectiveness seems to be that exposure to food (cues) directly increases individual’s motivation to consume the food, even if the individual is not hungry (Berridge et al., 2010).

Moreover, some typical characteristics of obese individuals seem to further undermine the efficiency of the established behavioral tools. Compared to healthy-weight individuals, obese individuals seem to show an increased sensitivity to the rewarding nature of palatable food (Kenny, 2011), an increased attention to food cues (Castellanos et al., 2009), and a weakened inhibitory response in the context of food (Schag et al., 2013). This profile underscores the urgent need for other behavioral change strategies that do not rely on the established practices such as education or training of inhibitory control. The present report seeks to contribute to this emerging stream of literature that attempts to reduce the pull of the tempting food rather than building on its avoidance or its willful resistance (Jansen et al., 2015; MacLean et al., 2015).

One paradigm that uses exposure to unhealthy food to *reduce* subsequent consumption (and thus enhance healthy eating) is the pre-exposure procedure (Geyskens et al., 2008). More specifically, physical exposure to tempting food in a situation that discourages consumption improved subsequent resistance to food temptations (Geyskens et al., 2008; de Boer et al., 2015; Duh et al., 2016; Goddyn and Dewitte, 2017). The assumed mechanism accounting for the process underlying the pre-exposure effect is based on cognitive control theory. Cognitive control processes refer to the ability of the cognitive system to perform well at demanding tasks by gradually and flexibly adjusting its information processing to vary adaptively from moment to moment depending on current goals (Botvinick et al., 2001). We propose that the behavioral conflict resulting from the exposure to temptation in a context where its consumption is discouraged triggers cognitive control processes that reduce approach behavior to the food (Dewitte et al., 2009). When a situation with overlapping characteristics is subsequently encountered (e.g., a taste test where food with a similar taste is presented), the previously recruited cognitive control processes are easily accessible and reduce consumption.

The pre-exposure effect has showed promising effects in reducing the consumption of healthy-weight people (Geyskens et al., 2008; de Boer et al., 2015; Duh et al., 2016; Goddyn and Dewitte, 2017); however, it has never been tested in obese-weight people. Obese people have been shown to react differently to exposure to tempting food compared to healthy-weight people in two ways. Compared to healthy people, they have been found to perform worse in restraining tasks as well as to value tempting rewards more (Nederkoorn et al., 2006a,b; Weller et al., 2008). The increased vulnerability to food cues and reduced inhibitory capacity of obese people makes it particularly challenging to expose them to high-caloric food items without inducing them to consume, which is essential in the pre-exposure procedure. Should they succeed, these two characteristics make it also difficult to produce a successful transfer to the subsequent tempting food situation. Nevertheless, recent evidence on control-readiness suggests that especially people with obesity can benefit from a paradigm in which self-control processes are activated during a pre-treatment phase to facilitate self-control in a subsequent decision situation (Kleiman et al., 2016). The objective of this paper is to explore the applicability of the pre-exposure procedure to reduce consumption of unhealthy food after exposure to similar tempting food in obese people. We expect that pre-exposure in tempting food cues will reduce subsequent food intake in healthy-weight as well as obese-weight individuals.

## Materials and Methods

### Participants and Design

The study took place in two different university labs (one in Belgium and one in Greece). Participants were recruited via announcements in social media and university database channels. The study was advertised as a taste assessment of food products. Our target was to compare 40 people with obese weight and compare them to a similar sample of healthy-weight participants. Forty was toward the higher end of previously used sample sizes in lab studies showing pre-exposure (e.g., Geyskens et al., 2008). The recruitment efforts did not lead to enrolment of high number of obese-weight participants. We reasoned that there were too few obese-weight people in the student town Leuven. Therefore, we continued our recruitment in Greece in a much bigger town where the obesity prevalence would be closer to the national average, which was 17% for Greece (compared to 18.6 in Belgium; OECD, 2017). The participation fee was 20 euros for Belgium and 10 for Greece.

One hundred and thirty-one individuals participated in the experiment (77% women, *M*_age_ = 27.55, *SD* = 9.45). Sixteen participants were not included in the analysis. Fourteen had a BMI between 25 and 30 while one had a BMI under 18. One participant did not follow the instructions of the experiment as she did not try the products in the taste task as she was instructed. There were no other exclusion criteria used. Our final sample was composed of 115 participants, 77 healthy-weight (*M*_BMI_ = 21.46, *SD* = 1.55) and 38 obese participants (*M*_BMI_ = 32.70, *SD* = 2.17).

A between-subject design was used with pre-exposure manipulation and weight status (normal vs. obese) as independent factors. Participants were randomly assigned to one of the pre-exposure conditions.

### Materials and Procedure

#### Pre-exposure Manipulation

After answering a question on how hungry they were (on a seven-point scale), participants were informed that they were going to participate in an unrelated word fluency task. They were randomly assigned to either a candy scrabble game (pre-exposure condition; PE) or to a foam scrabble game (control condition; CTR; Grubliauskiene and Dewitte, 2014). The letter candies had been identified as attractive by prior research (Grubliauskiene and Dewitte, 2014). Both groups received 30 letters (Haribo© candy letters in PE; letters made out of foam in CTR) and were instructed to form words using the letters to form words in the allotted time of four minutes. Words could also be formed in a cross-pattern (left-right, top-bottom). The experimenter took a picture and then removed the letters.

#### Taste Test

Participants received two bowls of the same volume of a tempting snack. We presented the test as a comparative taste test of brand products vs. their private label counterpart. The samples were named sample A and B. In actuality, both of the bowls contained the same product. They either received either two bowls of peanut M&M’s (600 g per bowl) or two bowls of Maltesers (400 g per bowl) in Belgium (depending on their preference) and two bowls Maltesers in Greece (200 g per bowl)^1^. To account for the product differences, we standardized consumption per product before analysis.

They were allowed to eat as much of the food as they wanted. Participants got questions like “How crunchy are these chocolate candies/crunchy nuts?,” “to what extent do these chocolate candies/crunchy nuts melt in your mouth,” etc., on both the brand and the private label. After they finished this task, the bowls were weighted. Participants were debriefed and were free to leave. The total procedure took between 20 and 30 min.

The distribution of the consumption volume (g) was skewed. To avoid an undue impact of large observations, we first log-transformed the consumption amount. We then standardized the log-transformed data to correct for the physical product differences.

## Results

### Demographics and Randomization Check

There were differences in the sample with regard to the age [*F*(1,114) = 7.72, *p* = 0.006, $\eta_{p}^{2}$ = 0.057] and the proportion of genders [χ^2^(1,114) = 13,168, *p* < 0.001, β = 1.852]. However, there was no difference in the average BMI [*F*(1,114) = 0.143, *p* = 0.705, $\eta_{p}^{2}$ = 0.001, see **Table 1**]. Furthermore, there were no differences in the age and BMI distribution across the conditions (*F*_age_ = 0.055, *p* = 0.815; *F*_BMI_ = 0.339, *p* = 0.561). There was no difference in the hunger levels between healthy- and obese-weight participants [*F*(1,114) = 0.07, *p* = 0.793, $\eta_{p}^{2}$ = 0.001]. Within the obese-weight group, there was no significant difference in the hunger levels between the CRT and PE conditions [*F*(1,114) = 0.87, *p* = 0.357, $\eta_{p}^{2}$ = 0.027]. Within the healthy-weigh group, there was a marginally significant difference [*F*(1,114) = 2.933, *p* = 0.091, $\eta_{p}^{2}$ = 0.038].

**Table 1:** Demographics.

	Belgium	Greece
Age	*M* = 30.15, *SD* = 10.88	*M* = 24.33, *SD* = 6.15
Women participants	93.4%	61.1%
BMI	*M* = 25.25, *SD* = 5.29	*M* = 25.11, *SD* = 5.98

### Main Analysis

**FIGURE 1:**
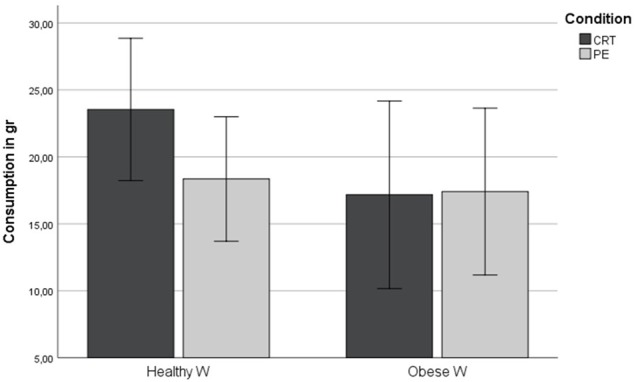
Mean consumption in the taste test.

We ran a two-way between-subject ANOVA with quantity consumed (standardized log data) as dependent variable and pre-exposure to tempting food and weight status as independent variables. **Figure 1** displays the results (with the untransformed variable to increase readability and with the error bars representing standard error). In spite of what we expected, the main effect of the manipulation was not significant, while the main effect of weight status was marginally significant [*F*(1,114) = 3.645, *p* = 0.059, $\eta_{p}^{2}$ = 0.032]. An unexpected significant interaction of weight status and the pre-exposure manipulation emerged [*F*(1,114) = 5.036, *p* = 0.027, $\eta_{p}^{2}$ = 0.044]. The interaction effect remained significant also after controlling for hunger levels [*F*(1,113) = 5.48, *p* = 0.021, $\eta_{p}^{2}$ = 0.050]. Furthermore, the marginally significant difference in the hunger levels across the two conditions found for the healthy-weight participants cannot account for the results as in the PE condition (where healthy-weight participants ate less) hunger levels where higher (*M* = 3.40, *SD* = 1.72) than in the CRT condition (*M* = 2.76, *SD* = 1.48).

To follow up the interaction effect, we conducted contrast analyses (simple main effects). We first tested the effect of the PE manipulation in both weight groups separately, and then tested the difference between the weight groups in the two PE conditions. In healthy-weight participants, the group that performed the scrabble task with candy letters (PE) ate significantly less during the subsequent taste test than the group that performed the scrabble task with foam letters [CTR, *F*(1,75) = 4.496, *p* = 0.037, $\eta_{p}^{2}$ = 0.057]. In obese-weight participants, both groups consumed a similar (low) amount of tempting snacks [*F*(1,37) = 1.565, *p* = 0.219, $\eta_{p}^{2}$ = 0.042]. Unexpectedly, in the control condition, the obese-weight participants ate significantly less than the healthy-weight participants [*F*(1,51) = 5.175, *p* = 0.021, $\eta_{p}^{2}$ = 0.103] but not in the experimental condition [*F*(1,66) = 0.096, *p* = 0.758, $\eta_{p}^{2}$ = 0.001, see **Table 2**). The results remained similar after controlling for hunger levels.

**Table 2:** Mean consumption in grams.

	Belgium (*N* = 61)		Greece (*N* = 55)	
	Healthy	Obese	Healthy	Obese
CRT	27.60 (*SD* = 21.06)	13.95 (*SD* = 12.59)	17.17 (*SD* = 7.38)	16.36 (*SD* = 16.13)
PE	16.82 (*SD* = 13.33)	16.46 (*SD* = 8.48)	19.68 (*SD* = 16.83)^1^	17.74 (*SD* = 15.69)

*^1^After standardizing and log-transforming the levels of consumption to account for the fact that the data were skewed the direction of the results changes. The levels of consumptions are higher in the CRT condition (M = 2.72, SD = 0.48) compared to PE condition (M = 2.64, SD = 0.82).*

### Robustness Check

Next, we wanted to test whether the fact that the study was conducted in two different countries (with different samples and slightly different operationalization in the measurement of the dependent variable) can have an impact on the results. Therefore, we ran several additional analyses to control for the differences. First, we conducted a multilevel analysis to test whether the nested structure of our data influences the results. The findings showed that the results are not influenced. The interaction effect remained significant (see **Table 3**). The interaction effect remained significant after running a two-way ANCOVA (CRT vs. PE condition X healthy- vs. obese-weight) including country sample as a control variable [*F*(1,113) = 4.982, *p* = 0.028, $\eta_{p}^{2}$ = 0.044]. Next, we ran a two-way ANCOVA including the product type as a control variable to test whether the use of different products for measuring consumption can influence the results. The interaction remained significant after controlling for the type of product used in the taste test [*F*(1,113) = 4.863, *p* = 0.030, $\eta_{p}^{2}$ = 0.043]. Last, the interaction remains significant when we ran a two-way ANCOVA controlling for the country sample, hunger levels, type of product, and age and gender differences [*F*(1,111) = 3.964, *p* = 0.049, $\eta_{p}^{2}$ = 0.039]. Finally, we could not find interactions between country, our manipulation, and weight group (see **Appendix 1**).

**Table 3:** Multilevel analysis results.

	*T*	*p*	β
Intercept	14.401	0.000	2.371
BMI	2.878	0.005	0.589
Pre-exposure condition	1.259	0.211	0.286
Interaction	-2.285	0.024	-0.636

*BMI: 0 = obese-weight and 1 = healthy-weight. Condition: 0 = CTR and 1 = PE.*

## Discussion

Previous research has demonstrated that the pre-exposure effect is a promising procedure to reduce consumption of unhealthy food (Geyskens et al., 2008; de Boer et al., 2015; Goddyn and Dewitte, 2017). The current study confirms this finding, but fails to replicate it in obese-weight participants. In healthy-weight subjects, this study replicates the pre-exposure effect with an adapted procedure. By exposing people to candy and asking them to make a word puzzle with these candies, the task context discourages them to eat the candies. Based on cognitive control theory (Verguts and Notebaert, 2009), we speculate that the conflict between task compliance (“use all the letters to form words”) and the desire to consume candy recruits cognitive control processes that reduce the pull of the temptation, and subsequently spill over to the next consumption conflict.

The pre-exposure effect mechanisms bear similarity to the counteractive self-control procedure (Trope and Fishbach, 2000). Earlier research showed that exposure to temptations (e.g., pictures of pizza’s) triggered individual’s eating restriction goal, which led to lower subsequent consumption (Fishbach et al., 2003). This effect presupposes the existence of a eating restriction goal, which is not necessary for the pre-exposure effect. Instead, the pre-exposure treatment seems to reduce the accessibility of the eating goal (Geyskens et al., 2008), which is consistent with a cognitive control account of the effect.

Based on the literature, the main threats to a successful replication of this procedure among individuals of obese weight seemed to be a reduced resistance to temptation during the pre-exposure to temptation, and/or a reduced transfer of the cognitive control processes from the pre-exposure phase to the subsequent measurement phase (Schag et al., 2013). However, the main reason we failed to replicate the pre-exposure among obese-weight individuals seems not be because of these expected problems but because they ate remarkably little in the control condition. One possible explanation for this effect can come from the experimental procedure itself. The fact that the study took place in a lab and not in a more everyday setting might have primed a dieting goal in obese-weight participants (Papies, 2012) or triggered social concerns (Robinson and Field, 2015), which might have reduced their consumption as an effect. Compared to previous studies assessing reactivity to food cues our study is the only one to our knowledge showing increased reactivity in the obese-weight sample. However, previous studies used tasks that did not include real food consumption (e.g., Loeber et al., 2012; Carters et al., 2015). More research is needed to further explore this consumption reduction in the control group of obese-weight participants, which may be promising in itself. Further adaptations to the procedure (e.g., repetitive pre-exposure trials; de Boer et al., 2015) may be in order to develop the pre-exposure procedure as a potential tool to reduce unhealthy snack consumption among obese patients.

The current study does not provide a conclusive test of the question as to if obese participants are sensitive to the pre-exposure procedure, as it has several limitations. First, the operationalization of the measurement of the depended variable was slightly different in the two countries. In Belgium, participants had to choose whether they will receive M&Ms or Maltesers for the taste test, while in Greece, all the participants received Maltesers. Furthermore, although we based ourselves on the cell size of previous demonstrations, the sample size of the obese participants is limited (*N* = 38) which may account for the fact that pre-exposure does not replicate in this part of the sample. In addition, the specific setting or task may be responsible for the lack of effect. It is therefore warranted to investigate if obese participants would react to other versions of the pre-exposure procedure, such as geometric puzzles with candy (Goddyn and Dewitte, 2017) or consumer knowledge testes (Duh et al., 2016). New studies would be informative with respect to the question if obese participants are sensitive to the pre-exposure procedure only if they eat at least as much as normal-weight participants in the control condition. Making the measurement phase more natural than a taste test (e.g., snacking when they watch movies) could be one possibility. Finally, our study did not include any direct measures of the activation of the cognitive control processes because we wanted to focus on the behavioral effect first. There is a possibility that cognitive processes were activated only for the healthy-weight part of our sample, and in this case, we may have to intensify the pre-exposure phase (e.g., more puzzles, more attractive sweets, and a pre-treatment increasing the behavioral conflict). Future endeavors can try to address all these limitations to ensure that the mechanism underlying pre-exposure effect has been activated for the entire sample. When such a studies have been done, we could start envisaging if and how the pre-exposure procedure could be used to complement existing therapies and for which type of obese people.

## Conclusion

The results show that the pre-exposure procedure is robust in healthy-weight subjects, but that the context *per se* already induced reduced consumption among obese-weight subjects. However, more research is needed to further explore the application of the pre-exposure procedure to reduce unhealthy snacking in obese-weight participants.

## Ethics Statement

This study was carried out in accordance with the recommendations of “KU Leuven ethical committee” with written informed consent from all subjects. All subjects gave written informed consent in accordance with the Declaration of Helsinki. The protocol was approved by the “KU Leuven ethical committee.”

## Author Contributions

AS gathered a part of the data, analyzed the results, and wrote a part of the manuscript. HG and AA gathered a part of the data and wrote a part of the manuscript. SD wrote a part of the manuscript.

## Conflict of Interest Statement

The authors declare that the research was conducted in the absence of any commercial or financial relationships that could be construed as a potential conflict of interest.

## Footnotes

1 We believe that the differences in the bowls offered in the two countries did not have an effect on the final results. The maximum consumption for Maltesers was 64.5 g. which is far from the total quantities offered in Greece (400 g). Furthermore, there was no significant difference in the consumption of Maltesers across countries [*F*(76) = 0.156, *p* = 0.686]. Actually, in Greece (where the bowls were smaller), participants ate slightly larger quantities (*M*_Greece_ = 18,13, *SD* = 14.33) than in Belgium (*M*_Belgium_ = 16.82, *SD* = 14.33).

## Appendix 1

**Table A1:** Country – condition interaction.

	*F*	*p*	$\eta_{p}^{2}$
Intercept	1490.189	0.000	0.931
Condition	0.683	0.410	0.006
Country	0.323	0.571	0.003
Interaction	0.700	0.405	0.006

**Table A2:** BMI – condition interaction.

	*F*	*P*	$\eta_{p}^{2}$
Intercept	1318.236	0.000	0.923
BMI	2.607	0.109	0.023
Country	0.130	0.719	0.001
Interaction	0.633	0.428	0.006
